# Interactions between HLA-B and leukocyte immunoglobulin like receptors B2 (LILRB2) correlate with HIV-1 disease outcomes

**DOI:** 10.1186/1742-4690-9-S2-P165

**Published:** 2012-09-13

**Authors:** E Martin Gayo, D Jones, F Pereyra, M Lichterfeld, RL Allen, XG Yu

**Affiliations:** 1 Ragon Institute of MGH, MIT and Harvard, Boston, MA, USA; 2 St George's, University of London, London, UK; 3 Infectious Disease Division, Massachusetts General Hospital, Boston, MA, USA

## Background

Recently, a genome-wide association study identified 3 amino acids (residues 67, 70, 97) in the HLA-B peptide binding groove that explain the classical HLA class I associations with HIV-1 immune control. However, how amino acid substitutions at these positions affect HIV-1 immune defense remains unclear. Leukocyte Immunoglobulin Like Receptors (LILR) are a class of immunoregulatory molecules that are expressed on myeloid dendritic cells and interact with HLA class I molecules as their physiologic ligands. Here, we assessed how different amino acids in the HLA-B binding groove may influence their binding to LILRs.

## Methods

Using a cell-free assay, the binding strength between 50 HLA-B alleles and recombinant LILRB2-Fc fusion protein was experimentally determined. Average bindings scores to LILRB2 were calculated for HLA-B allotypes sharing identical amino acid residues at position 67, 70 and 97. For comparison purposes, binding scores to LILRB1 were similarly determined. The average binding scores were then correlated to odds ratios reflecting the impact of the respective amino acids residues at position 67, 70 and 97 on HIV-1 disease progression.

## Results

Six amino acid variants at position 97, four amino acid variants at position 70, and five amino acid variants at position 67 were included into the analysis. Overall, we observed an almost perfect inverse correlation between the odds ratios of each amino acid variant at position 97 on HIV-1 control and LILRB2 binding scores of class I alleles carrying the respective amino acid variant (R=0.88, p=0.02). Similar findings were made for amino acid variants at position 70 (R=0.993, p=0.007) and position 67 (R=0.95, p=0.005). No significant correlations were observed when HLA-LILRB1 binding scores were used instead of LILRB2.

## Conclusion

These data suggest that amino acid polymorphisms at position 67, 70 and 97 of the HLA-B binding groove influence HIV-1 immune control through altered interactions with the immunomodulatory LILRB2 receptor.

